# Editorial: New insights into the treatment of aneurysms with flow diverters: novel indications and therapeutic advances

**DOI:** 10.3389/fneur.2023.1232777

**Published:** 2023-09-14

**Authors:** Yu Zhou, Jan-Hendrik Buhk, Jianmin Liu

**Affiliations:** ^1^Neurovascular Center, Changhai Hospital, Shanghai, China; ^2^Neuroradiology, Asklepios Hospitals St. Georg, Hamburg, Germany

**Keywords:** intracranial aneurysm, flow diverter, safety, indication, treatment advance

Flow diverters (FD) have originally been used primarily for large or giant unruptured aneurysms in the internal carotid artery. In recent years, the efficacy of such devices has led to their application in different circumstances, which include small or medium aneurysms, posterior circulation aneurysms, distal artery aneurysms, ruptured aneurysms, and traumatic aneurysms. However, while the use of FD in internal carotid unruptured aneurysms has been well-established, the usage in other scenarios remains a topic of debate. More information is needed for such applications. In addition, with the wide usage of FD in various aneurysms, new devices, novel therapeutic strategies, and approaches emerged, which simplify the usage of FD, and may have helped in reducing the complications. This Research Topic has collected a series of studies concerning such topics, i.e., FD out-of-the-box usage and therapeutic advances, to provide new insights into the treatment of aneurysms with FD.

The topic included 10 papers concerning different usages of FDs. Two of them were about the usage of FDs in the treatment of Blood Blister Aneurysms. Liu P. et al. introduced the results of 12 BBA patients in their single-center study, and Yan et al. studied the clinical and angiographic results of 13 recurrent BBAs. In both studies, all patients had favorable outcomes and satisfactory complete occlusion at the last follow-up. Two articles studied the application of FDs in small or medium aneurysms. Both Xie et al.'s and Li et al.'s articles showed that the treatment of small unruptured intracranial aneurysms with FD can be performed safely with satisfactory outcomes. Another six articles concentrated on posterior circulation aneurysms. Zhang et al. compared the safety of FD vs. conventional stent-assisted coiling in the treatment of vertebrobasilar dissecting aneurysms with intramural hematoma, basilar trunk and vertebrobasilar junction aneurysms, and intradural large vertebrobasilar artery aneurysms, respectively. Interestingly, all of them indicated a potential superiority of FD over conventional stents due to fewer complications and their effect in reducing intramural hematoma size. In the remaining three articles, Wang et al. introduced their experience of FD implantation in 16 patients with basilar artery aneurysms, which also indicated that FD is feasible and relatively safe in selected patients. Among 16 included patients, three experienced procedural complications (18.8%), including two ischemic strokes and one hydrocephalus, with resultant mortality in one case (6.3%). Median follow-up was 7.7 months and was available for 15 aneurysms. Complete/near-complete occlusion was seen in 13 (86.7%) aneurysms. A favorable result was also observed in Lu et al.'s study with 17 unruptured vertebral artery dissecting aneurysms. Xu et al. compared the results of FDs in anterior and posterior circulations and concluded that anterior circulation fusiform aneurysms have a lower occlusion rate than posterior circulation fusiform aneurysms.

In addition, another seven articles concerning new devices and treatment strategies were included. Liu X. et al. conducted a meta-analysis to evaluate the feasibility and safety of flow diversion in the treatment of intracranial aneurysms via the trans-radial approach and concluded that the trans-radial approach has a higher success rate and lower access-related complication rate. *Nan Li* introduced a new method, i.e., staged flow diverter implantation, to prevent delayed aneurysm rupture after FD treatment for large or giant aneurysms. In 30 patients with morphologies prone to rupture, no delayed aneurysm rupture occurred. Specifically, although Leo Bay and Neuroform atlas stent are not conventional FDs as we mentioned, the authors mentioned their flow diversion effects, and hence two articles by Duan et al. and Dong et al. were also included.

From the included studies in this Research Topic, it seems FDs are safe and effective in various off-label aneurysms, such as BBA, posterior circulation aneurysms, and small or medium aneurysms. These studies provide new insight into the treatment of aneurysms with FD as we anticipated. However, it also must be noted that patient bias existed in these studies, and overall, the complication rate was not eligible in posterior circulation aneurysms. FD should be chosen only for selected patients. In the future, more studies are needed to explore strategies, such as individual antiplatelet regimens, to decrease the potential complications.

## Author contributions

YZ and JL competed the draft. J-HB revised the manuscript extensively. All authors contributed to the article and approved the submitted version.

